# Ubiquitin C-hydrolase L1 as a cerebrospinal fluid and blood biomarker for prion diseases

**DOI:** 10.1093/braincomms/fcag363

**Published:** 2026-10-06

**Authors:** Sezgi Canaslan, Matthias Schmitz, Anna-Lisa Fischer, Susana Da Silva Correia, Angela Da Silva Correia, Tayyaba Saleem, Peter Hermann, Inga Zerr

**Affiliations:** Department of Neurology, University Medical Center Göttingen and the German Center for Neurodegenerative Diseases (DZNE), Göttingen 37075, Germany; Department of Neurology, University Medical Center Göttingen and the German Center for Neurodegenerative Diseases (DZNE), Göttingen 37075, Germany; Department of Neurology, University Medical Center Göttingen and the German Center for Neurodegenerative Diseases (DZNE), Göttingen 37075, Germany; Department of Neurology, University Medical Center Göttingen and the German Center for Neurodegenerative Diseases (DZNE), Göttingen 37075, Germany; Department of Neurology, University Medical Center Göttingen and the German Center for Neurodegenerative Diseases (DZNE), Göttingen 37075, Germany; Department of Neurology, University Medical Center Göttingen and the German Center for Neurodegenerative Diseases (DZNE), Göttingen 37075, Germany; Department of Neurology, University Medical Center Göttingen and the German Center for Neurodegenerative Diseases (DZNE), Göttingen 37075, Germany; Department of Neurology, University Medical Center Göttingen and the German Center for Neurodegenerative Diseases (DZNE), Göttingen 37075, Germany

**Keywords:** Creutzfeldt–Jakob disease, prion diseases, biomarker, ubiquitin C-hydrolase L1

## Abstract

Digital enzyme-linked immunosorbent assays are based on single-molecule array technology. In this study, ubiquitin C-terminal hydrolase L1 (UCH-L1) levels were measured in cerebrospinal fluid (CSF) and blood samples (serum or plasma) obtained from patients with sporadic Creutzfeldt–Jakob disease and fatal familial insomnia using a commercially available UCH-L1 assay. We determined the inter- and intra-assay variability as well as the stability of UCH-L1 detection to ensure a reliable assay performance. Cut-off values were calculated to differentiate prion disease patients from neurodegenerative and non-neurodegenerative control groups. Patients with Creutzfeldt–Jakob disease exhibited significantly higher levels of UCH-L1 in both CSF and serum than controls. Receiver operating characteristic (ROC) analysis yielded area under the curve (AUC) values ranging from 0.91 to 0.97 for CSF and from 0.58 to 0.86 for serum. These values allow an excellent discrimination between Creutzfeldt–Jakob disease patients and controls. Similar findings were observed in the fatal familial insomnia cohort. Here, ROC analysis indicated AUC values between 0.79 and 0.91 for CSF and up to 0.95 for plasma when distinguishing fatal familial insomnia patients from neurodegenerative, non-neurodegenerative and healthy controls. Altogether, our study suggests that detection of UCH-L1 levels in CSF and blood is suitable for the diagnosis of prion diseases.

## Teaser

Canaslan *et al*. evaluated ubiquitin C-hydrolase L1 (UCH-L1) as a biomarker for prion diseases using the highly sensitive single-molecule array platform. Elevated UCH-L1 levels in CSF and blood accurately distinguished sporadic Creutzfeldt–Jakob disease and fatal familial insomnia patients from controls, supporting blood-based UCH-L1 as a promising diagnostic biomarker.

## Graphical Abstract

**Graphical Abstract:**
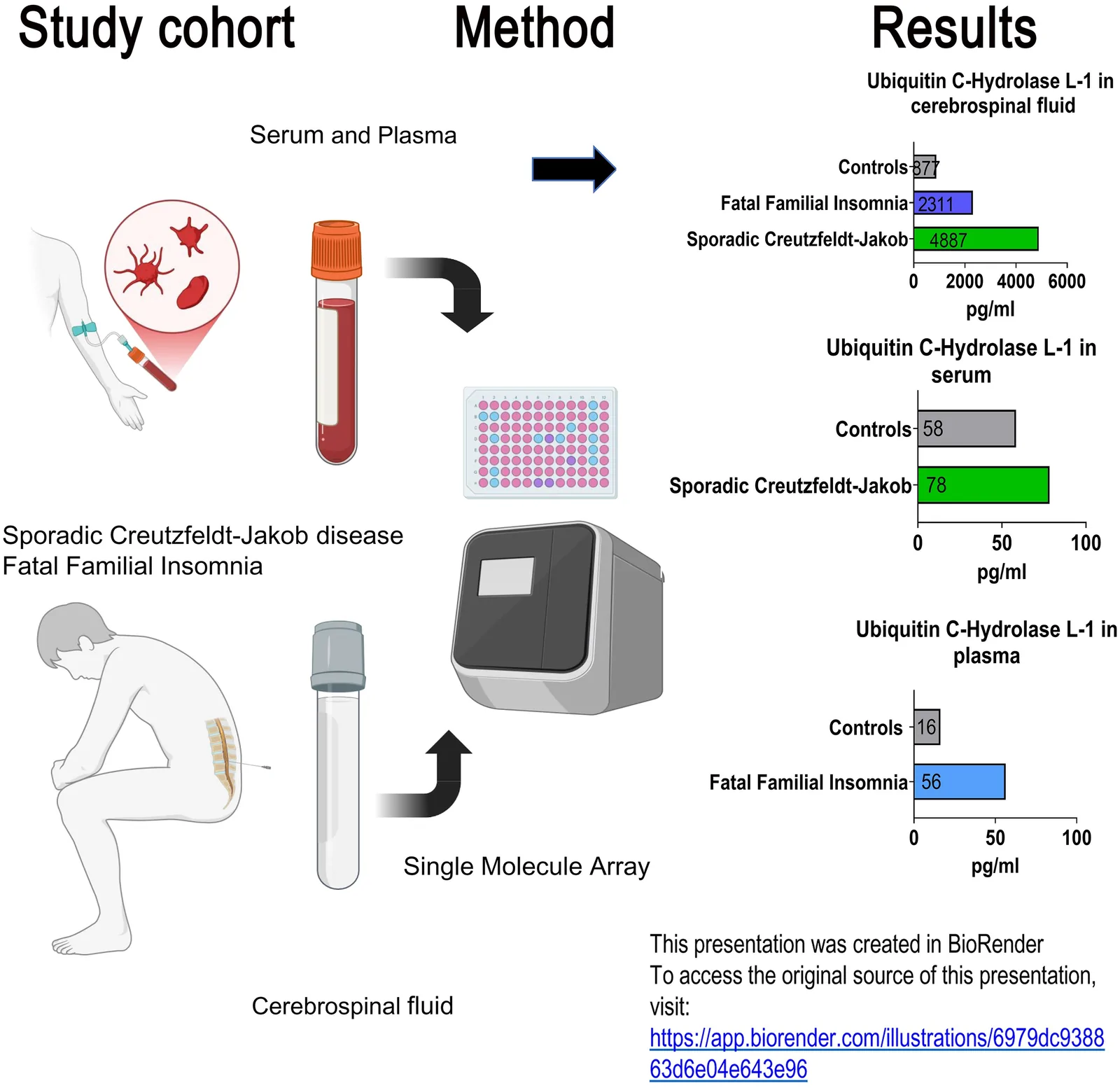

## Introduction

Human prion diseases consist of Creutzfeldt–Jakob disease, fatal familial insomnia and Gerstmann–Sträussler–Scheinker syndrome and include sporadic, genetic and acquired forms. They are caused by the conversion and aggregation of cellular prion protein (PrP^C^) into a neurotoxic conformer, called PrP scrapie (PrP^Sc^).^1^ Accumulation of PrP^Sc^ in the brain leads to protein aggregates that disrupt neuronal function and cause cell death.^1^ Characteristic spongiform changes develop due to neuronal loss and vacuolation. Symptoms include rapidly progressive dementia, ataxia and behavioural changes as neurons degenerate.

Currently, a number of accurate surrogate diagnostic markers, such as 14-3-3, tau, alpha-synuclein and PrP^Sc^ detection via real-time quaking-induced conversion (RT-QuIC) in cerebrospinal fluid (CSF), have been applied for sporadic Creutzfeldt–Jakob disease diagnosis.^2-7^ Diagnosing fatal familial insomnia at an early clinical stage poses significant challenges due to the considerable clinical variability and lack of easily applicable biomarkers of the disease. CSF biomarkers, including 14-3-3 and tau, are typically negative, and even PrP^Sc^ detection via RT-QuIC in CSF can fail in a substantial number of patients (test sensitivity of 28% only^3,8^). Even though lumbar puncture is potentially invasive, CSF is used as a common matrix for routine diagnostics, since the development of blood tests is more challenging and more difficult to standardize. To date, there is no blood-based test for routine prion disease diagnosis available.

Ubiquitin carboxyl-terminal hydrolase L1 (UCH-L1) is a highly expressed and abundant enzyme in the central nervous system.^9-12^ Given its involvement in the ubiquitin-proteasome system (UPS), UCH-L1 functions primarily as a de-ubiquinating enzyme, which is essential for protein degradation and turnover within neurons.^13^ Its dysfunction is linked directly to neurodegenerative proteinopathies by enabling pathological accumulation of proteins due to impaired degradation, leading to cellular stress, synaptic dysfunction and neuronal loss.^14^ UCH-L1 is found in protein inclusions in some neurodegenerative diseases, like Lewy body dementia or Pick disease.^15^ PrP^Sc^ aggregates, which are resistant to proteasomal degradation, are hallmarks of prion diseases.^16,17^ The functionality of the UPS and the level of ubiquitinated proteins are also affected in the presence of toxic protein aggregates.^18^ As UCH-L1 has a role in the clearance of aggregates and in maintaining the free-ubiquitin pool for further ubiquitination, UCH-L1 can affect or be affected by the disease condition.

For an ultrasensitive detection of UCH-L1, in particular in blood, we applied the digital protein detection system single-molecule array (SIMOA). The advantage is that SIMOA enables the detection of femtomolar protein amounts in a sample, which are often not measurable with previous analogue immunodetection methods, such as colorimetric ELISA.^19^ This study examined UCH-L1 levels in CSF and blood of sporadic Creutzfeldt–Jakob disease and fatal familial insomnia patients using SIMOA-based assays. In the sporadic Creutzfeldt–Jakob disease cohort, we assessed the influence of *PRNP* codon 129 polymorphism and PrP type on UCH-L1 levels, evaluated diagnostic accuracy using receiver operating characteristic (ROC) analysis and tested assay performance through inter- and intra-assay variability analyses.

## Materials and methods

### Compliance with ethical standards

Studies involving human biofluid samples were approved by the local ethics committee of University Medicine Göttingen, Von-Siebold-Str. 3, 37075 Göttingen (approval no. 11/11/93; prion diseases). Control samples were obtained through other observational studies, which were also approved by the local ethics committee of the University Medical Center Göttingen (approval nos. 19/11/09 and 9/6/08).

#### Samples

A possible diagnosis is based on progressive dementia with typical clinical features, short disease duration and no alternative cause, according to the consensus criteria, as described before.^20,21^

The information about patient cohort is found in Table 1 and Supplementary Table 1. The analysis of the codon 129 MV genotype of *PRNP* was performed after the isolation of genomic DNA from blood samples according to standard methods.^22^ The molecular PrP^Sc^ types (1 and 2) correlate with clinicopathological phenotypes of sporadic Creutzfeldt–Jakob disease. They were determined by western blot analysis of protease-resistant prion protein in brain tissue. After proteinase K digestion, the unglycosylated PrP^Sc^ fragment shows distinct electrophoretic mobilities at ∼21 kDa (Type 1) and 19 kDa (Type 2).

**Table 1:** Demographic characteristics of the prion disease patient cohort and UCH-L1 concentrations in CSF, plasma and serum

	Sporadic Creutzfeldt–Jakob disease		Fatal familial insomnia	
	CSF	Serum	CSF	Plasma
Age (±SD)	69.47 ± 9.5	69.31 ± 8.9	51.8 ± 13.9	52.62 ± 12.8
*N*, Sex (M/F)	80 (44/36)	33 (21/12)	14 (8/6)	30 (16/14)
UCH-L1 (pg/ml) (±SEM)	4887 ± 440	78.42 ± 8.9	2311 ± 426	56.35 ± 9.2

Age- and gender-matched control cases are neurological controls without neurodegeneration, such as psychiatric disorders, epilepsy, vertigo, pain syndromes, headache and peripheral neuropathies for CSF and serum samples. Neurodegenerative controls derived from Alzheimer’s disease patients and synucleinopathies patients were diagnosed according to the established criteria^23,24^ (Supplementary Table 1).

Plasma samples are from healthy controls (blood donors), Department for Transfusion Medicine, University Medical Center. Depending on the availability of serum or plasma samples, we have used plasma or serum in the analysis, keeping the same fluid for the particular disease. Additionally, the availability of matching samples was considered. If sufficient material was available from matching samples, these samples were included.

### Determination of UCH-L1 levels

UCH-L1 concentrations in CSF, serum and plasma were quantified using SIMOA assays (Quanterix, MA, USA) on the SR-X platform according to the manufacturer’s protocol. Prior to analysis, all samples and reagents were allowed to equilibrate to room temperature. CSF and serum samples were briefly vortexed, and serum samples were subsequently centrifuged at 10 000 rpm for 5 min to remove particulate material. CSF and serum samples were diluted 1:10 and 1:4, respectively. Diluted samples were mixed with bead solution (25 µl) and detector antibody solution (20 µl) and incubated at 35°C with shaking at 800 rpm for 35 min. After the washing steps, 100 µl of streptavidin-β-galactosidase solution was added and incubated under the same conditions for 10 min. Following a final wash and a 10-min drying step, the plates were analysed using the SR-X instrument.

#### Statistical analysis

Comparisons of more than two groups were performed using one-way ANOVA with Tukey’s *post hoc* test for parametric data or the Kruskal–Wallis test with Dunn’s correction for non-parametric data. Two-group comparisons used an unpaired Student’s *t*-test or Wilcoxon–Mann–Whitney test, as appropriate. Analyses were conducted in GraphPad Prism.

Statistical significance was defined as *P* < 0.05 (*), *P*  *<* 0.01 (**), *P*  *<* 0.001 (***) and *P* < 0.0001 (****).

## Results

### Assay variations of UCH-L1 in CSF, serum and plasma

The intra- and inter-assay variability of the UCH-L1 measurements was analysed across CSF, serum and plasma. Intra-assay variation was generally below 20% in serum (blue dots) and CSF (shell pink dots), with higher variability observed in some plasma samples (purple dots) (Fig. 1A). Inter-assay variation remained under 20% in most cases, with only four outliers exceeding this threshold (Fig. 1B).

**Figure 1:**
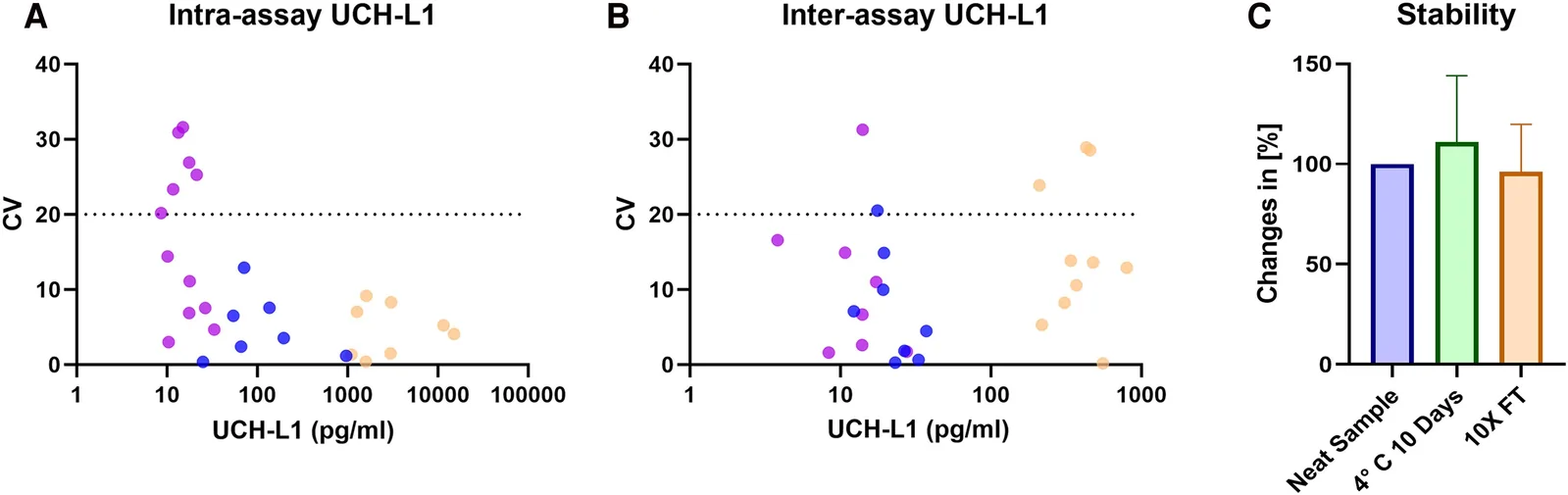
**Analysis of intra- and inter-assay variation of CSF and blood UCH-L1 under different conditions.** (**A**) Purple dots represent plasma, blue dots represent serum, and beige dots represent CSF. Intra-assay measurements of CSF (*n* = 8) and serum (*n* = 7) showed coefficients of variation (CV) below 20%, whereas some plasma samples (*n* = 12 total) showed CVs of up to ∼30%. (**B**) Inter-assay measurements of CSF (*n* = 10), serum (*n* = 8) and plasma (*n* = 8) samples showed CV mostly below 20%. (**C**) Marker stability of UCH-L1 was shown after 10 days at 4°C of incubation and after 10 freezing and thawing cycles (10× FT). CSF (*n* = 6), serum (*n* = 6) and plasma (*n* = 5) samples were analysed. Statistical analysis was performed using the Kruskal–Wallis test (*P* values > 0.05) [non-significant (NS); degrees of freedom: 2]. Data revealed a good reproducibility and stability of ubiquitin C-terminal hydrolase 1.

Afterwards, we investigated the stability of UCH-L1-detection under different conditions in different fluids, including storage at 4°C for up to 10 days and up to 10 freeze–thaw cycles between room temperature and −80°C. Our observations indicated that UCH-L1 levels remained stable under these conditions (Fig. 1C).

### UCH-L1 concentration in CSF of prion disease patients

CSF samples from patients with sporadic Creutzfeldt–Jakob disease, fatal familial insomnia and neurological control groups [neurodegenerative (ND) and non-neurodegenerative (non-ND)] were analysed using a commercial SIMOA-based UCH-L1 assay (Quanterix).

Fatal familial insomnia and sporadic Creutzfeldt–Jakob disease patients exhibited elevated CSF UCH-L1 levels compared with ND and non-ND controls (Fig. 2A). In contrast, no significant difference was observed between sporadic Creutzfeldt–Jakob disease and fatal familial insomnia patients (Fig. 2A). Subsequently, ROC curve analysis was performed to estimate the diagnostic accuracies. For sporadic Creutzfeldt–Jakob disease, the area under the curve (AUC) ranged from 0.91 to 0.97 depending on the control group used (Fig. 2B and D). For fatal familial insomnia, values between 0.79 and 0.91 were obtained (Fig. 2C and E), again depending on the comparison group. The discrimination was always better against non-ND controls than ND controls, reflected by higher AUC values. When both control groups were combined, an optimal cut-off of >2138 pg/ml was identified for sporadic Creutzfeldt–Jakob disease. At this threshold, CSF UCH-L1 achieved a sensitivity of 75.61% (95% CI: 65.31–83.62%) and a specificity of 96.21% (95% CI: 91.44–98.37%), with a likelihood ratio of 19.96. For fatal familial insomnia, a cut-off of >2200 pg/ml provided a sensitivity of 64.29% (95% CI: 38.76–83.66%) and a specificity of 96.97% (95% CI: 92.47–98.82%), with a likelihood ratio of 21.21.

**Figure 2:**
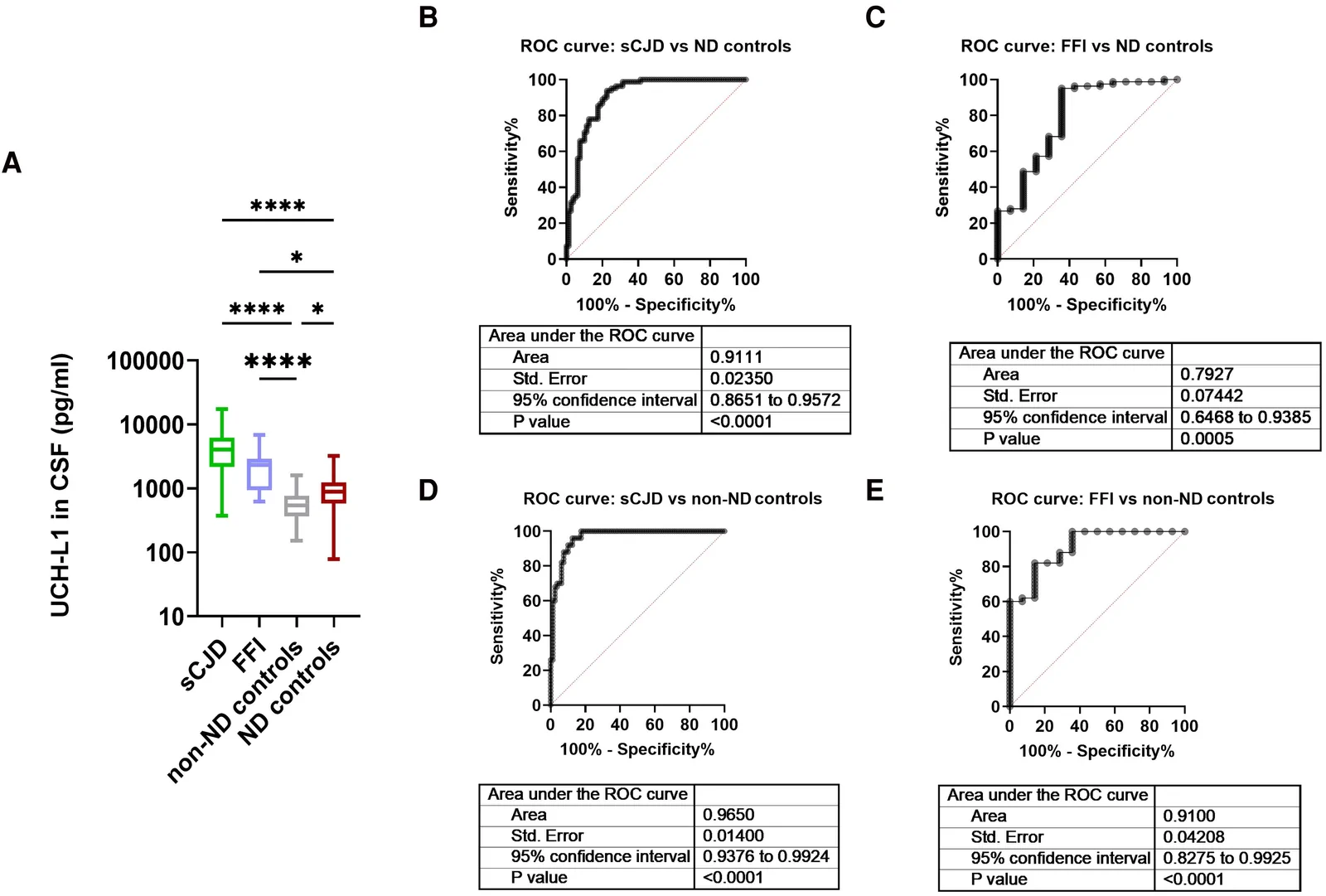
**Detection of UCH-L1 levels in CSF from prion disease and control groups.** (**A**) UCH-L1 levels in CSF were significantly elevated in sporadic Creutzfeldt–Jakob disease patients compared to controls [non-neurodegenerative controls (non-ND)]. The fatal familial insomnia group also exhibited higher CSF UCH-L1 levels compared to controls. (**B** and **C**) ROC curve analysis of CSF UCH-L1 demonstrated high diagnostic accuracy for distinguishing sporadic Creutzfeldt–Jakob disease and fatal familial insomnia from neurodegenerative (ND) controls, (*P* < 0.0001). (**D** and **E**) ROC curve analysis for differentiating sporadic Creutzfeldt–Jakob disease and fatal familial insomnia from non-ND controls showed an AUC of 0.91–0.96 (*P* < 0.0001). Sample sizes were as follows: sporadic Creutzfeldt–Jakob disease (*n* = 80), ND controls (*n* = 50), non-ND controls (n = 82) and fatal familial insomnia (*n* = 14). Mean values are indicated. Statistical analysis was performed using the Kruskal–Wallis test (*P* values indicated as *P < 0.05, **P < 0.01, ***P < 0.001; degrees of freedom = 3). Data suggested C-terminal hydrolase 1 as a potential CSF biomarker with a good diagnostic accuracy.

### Determination of UCH-L1 in blood of prion disease patients

When we measured UCH-L1 levels in blood samples (serum or plasma, depending on availability), we observed that serum UCH-L1 was significantly higher in sporadic Creutzfeldt–Jakob disease patients compared with non-ND controls (*P* < 0.0001; Fig. 3A). ROC curve analysis yielded AUC values between 0.58 and 0.86 when distinguishing sporadic Creutzfeldt–Jakob disease from ND and non-ND controls (Fig. 3B and C). At a cut-off of >65.39 pg/ml, serum UCH-L1 showed a sensitivity of 54.55% (95% CI: 37.99–70.16%) and a specificity of 71.33% (95% CI: 63.64–77.97%), with a likelihood ratio of 1.903 (poor discrimination).

**Figure 3:**
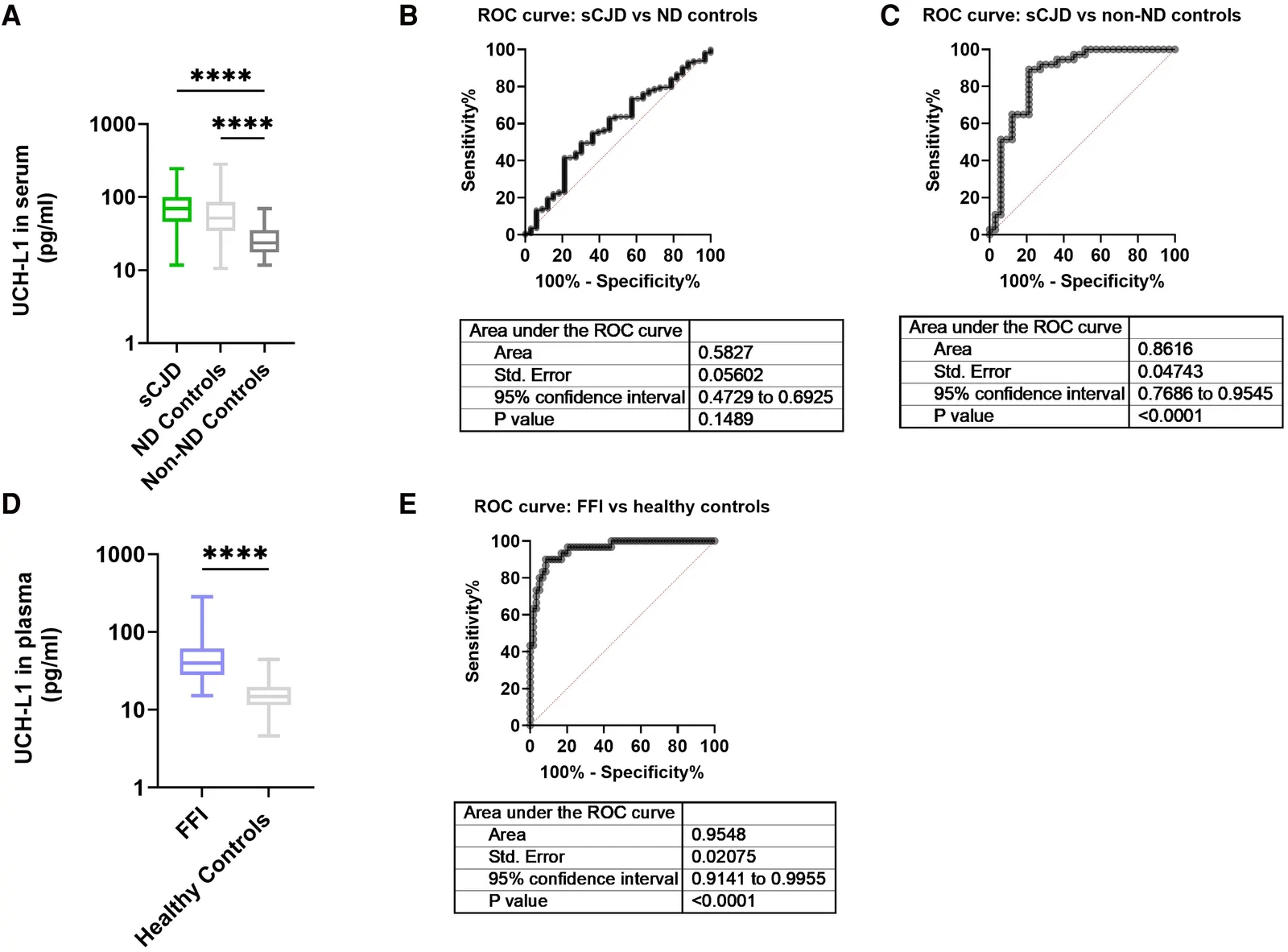
**Detection of UCH-L1 in blood from prion disease and control groups using SIMOA.** (**A**) Serum UCH-L1 levels were significantly elevated in sporadic Creutzfeldt–Jakob disease patients compared to controls [non-neurodegenerative (non-ND) and neurodegenerative (ND)]. (**B** and **C**) ROC curve analysis of serum UCH-L1 demonstrated an AUC of 0.58–0.86 (*P* < 0.0001) for distinguishing sporadic Creutzfeldt–Jakob disease from controls. (**D**) UCH-L1 levels in plasma were significantly elevated in fatal familial insomnia patients compared to controls [healthy controls (HC)]. (**E**) ROC curve analysis of plasma UCH-L1 showed an AUC of 0.95 (*P* < 0.0001) for differentiating fatal familial insomnia from HC. Sample sizes were as follows: sporadic Creutzfeldt–Jakob disease serum (*n* = 33), non-ND control serum (*n* = 37), ND control serum (*n* = 113), fatal familial insomnia plasma (*n* = 30) and control plasma (*n* = 59). Mean values are indicated. Statistical analysis was performed using the Wilcoxon Mann–Whitney test (*P < 0.05, **P < 0.01, ***P < 0.001; *Z* = 7.18). Data suggested C-terminal hydrolase 1 as a potential blood biomarker for sCJD and FFI diagnosis.

In plasma, UCH-L1 levels in fatal familial insomnia patients were significantly increased compared with healthy controls (*P* < 0.0001; Fig. 3D). ROC analysis showed an AUC of 0.95 (excellent discrimination) (Fig. 3E).

Using a cut-off of >25.97 pg/ml, plasma UCH-L1 achieved a sensitivity of 90% (95% CI: 74.38–96.54%) and a specificity of 91.53% (95% CI: 81.65–96.33%), with a positive likelihood ratio of 10.62 (strong discrimination).

### Impact of *PRNP* codon 129 MV genotype and type of PrP^Sc^

Subsequently, we were interested to know whether UCH-L1 levels in CSF and serum correlated with age at disease onset in fatal familial insomnia. However, we found no significant associations in either fluid (Supplementary Fig. 1A, B). Moreover, we analysed if UCH-L1 levels were influenced by *PRNP* codon 129 MV genotype or PrP^Sc^ subtype in Creutzfeldt–Jakob disease. No significant differences were detected across MM, MV and VV genotypes in CSF or serum, nor between PrP^Sc^ types 1 and 2 in CSF (Supplementary Fig. 2A–C). A non-significant trend towards lower UCH-L1 levels in MM patients was observed, which needs to be confirmed in larger cohorts.

## Discussion

The availability of biomarkers in more accessible body fluids than CSF is increasingly important for diagnosing neurodegenerative diseases. Beyond diagnosis, such biomarkers facilitate longitudinal monitoring of disease progression and therapeutic responses. Although established CSF biomarkers, including 14-3-3 and RT-QuIC, are widely used in prion diseases, additional biomarkers detectable in easily accessible body fluids would further improve Creutzfeldt–Jakob disease diagnosis.^3-5,25^ One potential candidate is UCH-L1, which is a deubiquitinating enzyme, highly expressed in the central nervous system, accounts for 1–5% of soluble proteins in the brain, and it is mainly localized in neurons.^11,12^

In our study, we measured UCH-L1 levels in CSF and blood of sporadic Creutzfeldt–Jakob disease and fatal familial insomnia patients, compared to ND-, non-ND- or healthy controls, depending on availability. To increase the sensitivity of our assay, in particular for blood analysis, we used assays based on ultrasensitive SIMOA technology.

The marked neuropathological differences between sporadic Creutzfeldt–Jakob disease and fatal familial insomnia suggest that increased UCH-L1 levels are unlikely to be directly driven by PrP^Sc^ aggregation itself.^26,27^ Instead, UCH-L1 elevation may reflect a common downstream mechanism, such as acute neuronal injury associated with neuroinflammation. Microglial activation is a prominent and early feature of human prion diseases and is often more intense than in other neurodegenerative disorders, possibly related to their rapid and aggressive clinical course.^28^ Activated microglia can induce neuronal damage through cytokine release, oxidative stress and excitotoxic pathways, independent of spongiform change. This mechanism could explain elevated UCH-L1 in fatal familial insomnia, where neuronal loss and gliosis predominate despite minimal PrP^Sc^ deposition.^29^ Supporting this interpretation, increased UCH-L1 has also been reported in multiple sclerosis in association with neuroinflammatory activity rather than protein aggregation.^30^

Our data revealed a significant increase in both biological fluids (CSF and blood) of UCH-L1 levels in sporadic Creutzfeldt–Jakob disease patients compared to controls (ND and non-ND). To investigate the diagnostic accuracy, we performed ROC curve analysis of UCH-L1 regulation in our patient cohort. Interestingly, independent of the type of control (ND versus non-ND), we obtained AUC values of 0.91 and 0.97 for CSF and 0.58 and 0.86 for serum UCH-L1. This indicates a good diagnostic accuracy of UCH-L1 for sporadic Creutzfeldt–Jakob disease diagnosis compared to commonly used markers, such as 14-3-3, tau, alpha-synuclein or RT-QuIC.^2,5,31,32^ For CSF UCH-L1, the optimal cut-off value at >2138 pg/ml results in a sensitivity of 75.61% and a specificity of 96.21%.

The regulation of UCH-L1 in sporadic Creutzfeldt–Jakob disease/fatal familial insomnia is independent of disease onset and severity (fatal familial insomnia), the kind of *PRNP* codon 129 MV genotype or subtype in sporadic Creutzfeldt–Jakob disease which is an advantage of this marker compared to other surrogate markers, such as 14-3-3.^33^ Another important aspect is the regulation of UCH-L1 in the serum of Creutzfeldt–Jakob disease patients. Commonly used surrogate markers for CSF, such as 14-3-3 or alpha-synuclein, exhibit no diagnostic relevance in blood.^31,34^ Although the AUC value in serum is lower than in CSF, we consider UCH-L1 a potentially promising marker for the serum diagnosis of sporadic Creutzfeldt–Jakob disease in particular for differentiation from non-ND controls. Compared to other blood biomarkers for sporadic Creutzfeldt–Jakob disease, such as plasma NfL, beta-synuclein and total tau, which, depending on the patient cohorts, show comparable AUCs values of around 0.90–0.93 for distinguishing sporadic Creutzfeldt–Jakob disease from controls.^35,36^

In addition to prion diseases, elevated UCH-L1 levels had been observed in CSF and serum of patients with amyotrophic lateral sclerosis.^37^ However, the sensitivity (61.43%) and specificity (79.59%), along with the proposed cut-off value for serum UCH-L1 (>15.22 pg/ml), were lower than those reported for prion diseases (>65.39 pg/ml). When additional neurodegenerative disease controls were included in our cohort, the sensitivity and specificity decreased further to 54.55 and 71.33%, respectively, indicating only moderate diagnostic performance.

Since fatal familial insomnia is rarer than Creutzfeldt–Jakob disease and has fewer established diagnostic markers, the observed upregulation of UCH-L1 in fatal familial insomnia represents a particularly intriguing finding. Although fatal familial insomnia is a genetic prion disease, reliable biomarkers for disease onset and progression are still lacking. Most surrogate CSF markers yield negative results, and RT-QuIC shows lower sensitivity in fatal familial insomnia compared to sporadic Creutzfeldt–Jakob disease.^3,8^ UCH-L1 upregulation in CSF in fatal familial insomnia was similarly reflected in plasma. AUC values ranged from 0.79 to 0.91 in CSF and reached 0.95 in plasma, depending on the control group. Discriminatory performance was highest versus healthy controls and lowest versus neurodegenerative disease controls, likely reflecting overlapping neuronal injury and glial activation in the latter group.

The study has several limitations. First, UCH-L1 was measured in serum for sporadic Creutzfeldt–Jakob disease and in plasma for fatal familial insomnia, which may introduce minor variability between sample types. Additionally, the healthy plasma control group in the fatal familial insomnia comparison suggests a lower impact than the neurological control group. However, we expect a similar level of measurement accuracy in both groups because UCH-L1 levels were not significantly different in both types of prion diseases. No data are available on the diseases duration of our cohort.

Moreover, the results require validation in a second independent cohort to confirm their reliability. Finally, the underlying mechanism of UCH-L1 regulation remains speculative. It may reflect neuronal damage or represent a potential neuroprotective response. However, sporadic Creutzfeldt–Jakob disease progresses rapidly and irreversibly, so even a sudden increase in UCH-L1 may be insufficient to halt the disease. UCH-L1 could also be sequestered within proteasome-resistant prions, prompting cells to upregulate its production and resulting in extremely high levels.

In conclusion, we reported the upregulation of UCH-L1 levels in CSF and blood of sporadic Creutzfeldt–Jakob disease and fatal familial insomnia patients, showing a reliable diagnostic accuracy. In particular, the detection of UCH-L1 in blood is consistent with its detection in CSF.

Blood-based UCH-L1 assessment may serve as a valuable tool in future follow-up or treatment studies to evaluate therapeutic efficacy, particularly when lumbar puncture is not feasible or when regular longitudinal sampling is required.

## Supplementary Material

fcag363_Supplementary_Data

## Data Availability

The datasets generated during the current study are available from the corresponding author upon reasonable request. Raw data are available under https://doi.org/10.6084/m9.figshare.32159049. Data were analysed using GraphPad Prism software, and no custom code or scripts were used.
