# Supplementary material for: Ubiquitin C-hydrolase L1 as a cerebrospinal fluid and blood biomarker for prion diseases
Source: Brain Commun. 2026 Oct 6;8(5):fcag363. doi: 10.1093/braincomms/fcag363 (PMC13663794; doi:10.1093/braincomms/fcag363)
Supplement: Supplementary file 1 [file fcag363_supplementary_data.pdf]

Supplementary Table 1: Demographic characteristics of the control cohort and UCH-L1 concentrations in CSF, plasma and serum.

	CONTROLS			CONTROLS NEURODEGENERATIVE	
	CSF$^{(Non-ND)}$	Serum$^{(Non-ND)}$	Plasma$^{(HC)}$	CSF$^{(ND)}$	Serum$^{(ND)}$
**AGE (± SD)**	64.65 ± 12.7	65.76±144	-	68.11 ± 11.4	69.09± 10.9
**N, SEX (M/F)**	50 (23/27)	37 (23/14)	59^{RU}	82 (21/18)^{RU}	113 (53/35)^{RU}
**UCH-L1 (± SEM)**	619 ± 49	27.96 ± 2.3	16.24 ± 0.93	1035 ± 68	68.31 ± 4.9

ND = neurodegenerative (Alzheimer's disease CSF, n=13; synucleinopathies CSF, n= 69; Alzheimer's disease serum, n= 53; synucleinopathies serum, n= 60; HC = healthy controls, RU = rest unknown

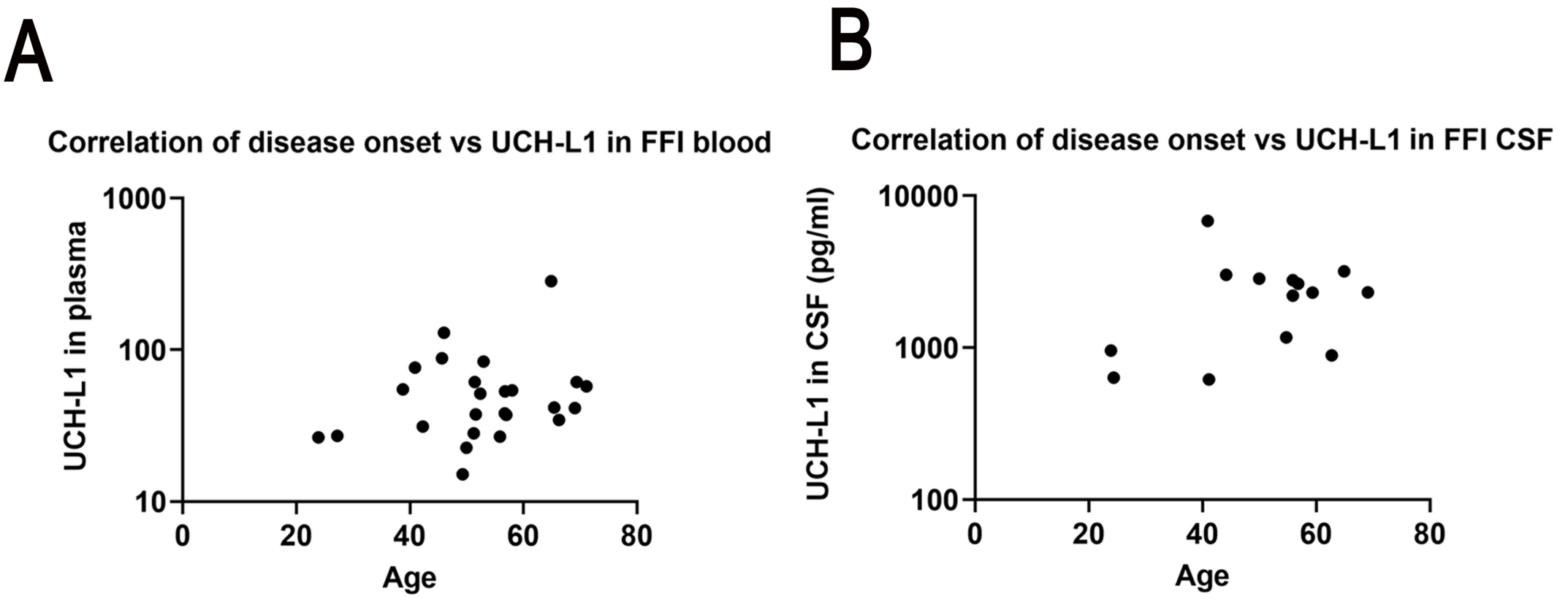

A

Spearman r	
r	0.2162
95% confidence interval	-0.2075 to 0.5716
P value	
P (two-tailed)	0.2994
P value summary	ns
Exact or approximate P value?	Approximate
Significant? (alpha = 0.05)	No
Number of XY Pairs	25

B

Spearman r	
r	0.2176
95% confidence interval	-0.3690 to 0.6802
P value	
P (two-tailed)	0.4542
P value summary	ns
Exact or approximate P value?	Exact
Significant? (alpha = 0.05)	No
Number of XY Pairs	14

Supplementary Figure 1: Correlation analysis of UCH-L1 levels in plasma and CSF of FFI patients with age at onset. A-B) Spearman correlation analysis of ubiquitin C-terminal hydrolase L1 (UCH-L1) levels in plasma and cerebrospinal fluid (CSF) of fatal familial insomnia (FFI) patients with age at onset revealed no significant association. (CSF n=14, plasma n=25).

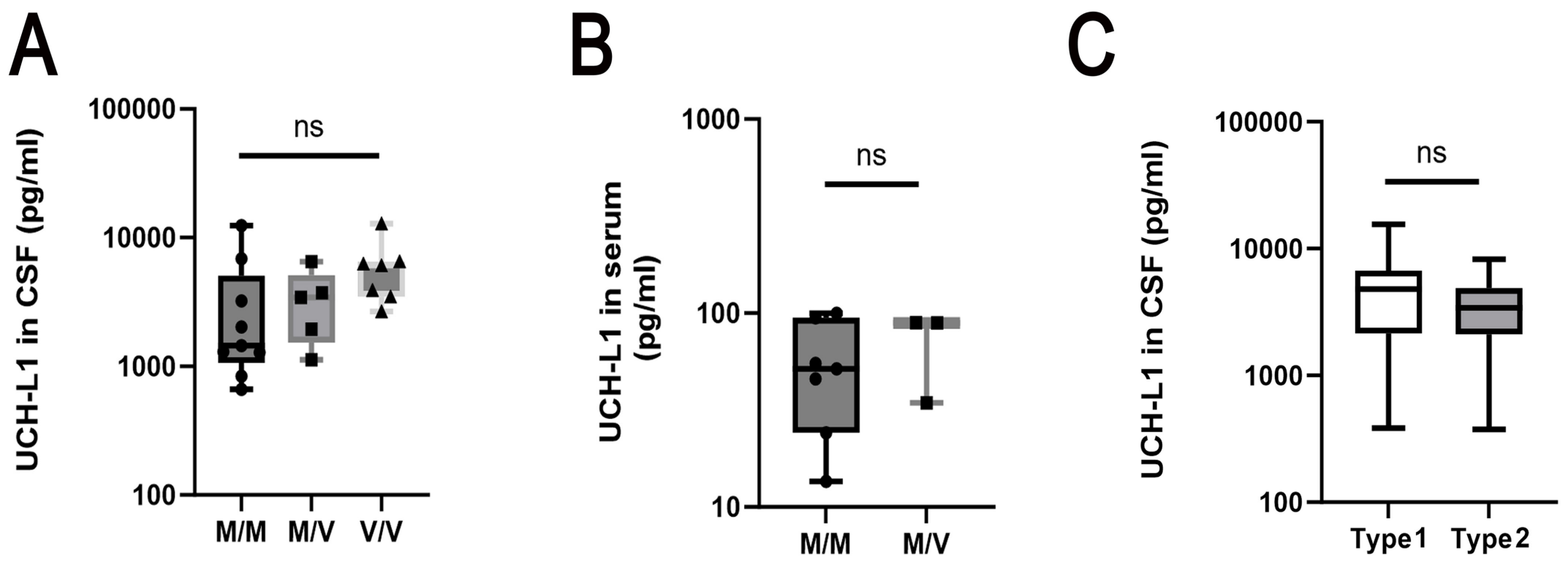

Supplementary Figure 2: Effect of genotype and PrP^{Sc} type on UCH-L1 levels in CSF and serum. A) Analysis of ubiquitin C-terminal hydrolase L1 (UCH-L1) levels across different sporadic Creutzfeldt-Jakob disease (sCJD) *PRNP* codon 129 MV genotypes (MM= 9, MV= 5, VV= 7) showed no significant differences in cerebrospinal fluid (CSF). B) Analysis of UCH-L1 levels across different sCJD *PRNP* codon 129 MV genotypes (MM= 7, MV= 3) showed no significant differences in serum. C) Analysis of UCH-L1 levels among different prion protein scrapie (PrP^{Sc}) subtypes [type 1 (n= 32) and type 2 (n=22] showed no significant differences in CSF. Mean values are indicated. Statistical analysis was performed using the Kruskal–Wallis test (p values indicated as $p > 0.05$ non-significant (ns); degrees of freedom = 2). Additionally, we applied the Wilcoxon Mann–Whitney-Test for the comparison of two groups.
